# Supplementary material for: A Computational Perspective on NeuroAI and Synthetic Biological Intelligence
Source: ArXiv. 2025 Oct 9:arXiv:2509.23896v2. Preprint. [Version 2] (PMC12632695)
Supplement: Supplement 1 [file NIHPP2509.23896v2-supplement-1.pdf]

Supplemental Tables

S1. Field Relations in the NeuroAI Hierarchical Graph

Field Name	Brief Description
Neuroscience (NS)	The study of the nervous system, including brain function, neural circuits, plasticity, and cognitive processes Squire et al. (2012).
Cell & Tissue Engineering (CTE)	Engineering and cultivating biological tissues and 3D organoids for research, therapeutic applications, and experimental neuroscience van Blitterswijk and De Boer (2022); Hofer and Lutolf (2021).
Bioelectronics (BE)	The integration of electronics with biological systems to develop neural interfaces, biosensors, and stimulation devices for bidirectional communication with neural tissue Willner and Katz (2006).
Artificial Intelligence (AI)	The development of algorithms and computational systems that simulate aspects of human intelligence, including learning, reasoning, and problem-solving.
Data Science (DS)	The extraction, analysis, and interpretation of large-scale data, including biomedical and neuroscience datasets, to uncover patterns and derive insights Russell and Norvig (2016).
Computational Biology (CB)	The application of computational methods to analyze, model, and simulate biological processes, systems, and data Waterman (2018).
Synthetic Biology (SB)	The synthesizing of new biological systems or the modification of existing ones to enable functions and properties not present in the system naturally Serrano (2007).
Tissue Culture Computational Modeling (TCCM = CTE + CB)	The integration of tissue engineering and computational biology to simulate, analyze, and design biological tissue structure, including neural tissues Montes-Olivas et al. (2019).
Neural Tissue Culturing (NTC = NS + CTE + SB)	Combining neuroscience, tissue engineering and synthetic biology to cultivate neural cultures with structured cytoarchitecture and functional neural activity Lokai et al. (2023).
Cognitive and Systems Neuroscience (CSN = NS + CB + AI)	The study of brain functions and neural systems using computational modeling, AI, and systems neuroscience to understand cognition, learning, and adaptation Gazzaniga (2009); Metzler (2012).
Neuroinformatics (NI = NS + CB + DS)	The intersection of neuroscience, computational biology, and data science to manage, analyze, and model complex neural and electrophysiological data Arbib and Grethe (2001).

Field Name	Field Description
Machine Learning (ML = AI + DS)	The intersection of AI and data science to develop algorithms that learn from data and improve performance without explicit programming Bishop and Nasrabadi (2006).
Neuromorphic Architecture (NMA = BE + NS)	The design of hardware that mimics the structure and function of biological neural networks, utilizing event-driven processing and in-memory computing Abdallah and Dang (2022).
Neural Tissue Modeling (NTM = NI + CSN + TCCM)	The use of neuroinformatics, cognitive systems neuroscience, and computational modeling to simulate neural cultures' structural and functional dynamics Montes-Olivas et al. (2019); Poli et al. (2019).
Neural Learning Models (NLM = CSN + ML)	AI models inspired by neuroscience, integrating symbolic reasoning and neural learning mechanisms to improve adaptability, interpretability, and cognitive-like processing. Also includes task-oriented models such as reinforcement learning and active inference Garcez and Lamb (2023); Wiering and Van Otterlo (2012); Friston et al. (2016).
Neural Tissue Interfacing (NTI = NTC + BE)	The combination of neural culture engineering and bioelectronics to develop interfaces for recording, stimulating, and communicating with neural tissues. Passaro and Stice (2021).
Neuromorphic AI (NMAI = NMA + ML)	AI algorithms inspired by the structure and function of biological brains, optimized for neuromorphic computing architectures to achieve efficient, event-driven learning Ivanov et al. (2022); Schuman et al. (2022).
Feedback-Driven Learning (FDL = NTI + NLM)	A framework integrating neural interfaces, stimulation, and cognitive neuroscience to enable adaptive, real-time feedback learning in neural tissues. Kagan et al. (2022); Robbins et al. (2024).
Reservoir Computing (RC = NTI + NI + ML)	The application of BNNs as computational reservoirs, leveraging their dynamic neural activity for real-time information processing and learning Cai et al. (2023); Smirnova et al. (2023a).
Neuro Digital Twin Modeling (NDTM = NTM + NSA + NMAI)	The development of a high-fidelity digital replica (digital twin) of neural tissue cultures and organoids, integrating neuro-symbolic AI and computational modeling for simulation and analysis Möller and Pörtner (2021).
Synthetic Biological Intelligence (SBI = FDL + RC)	The utilization of BNNs as adaptive learning systems, leveraging real-time feedback and reservoir computing to perform cognitive-like tasks Smirnova et al. (2023c).
NeuroAI (NeuroAI = NDTM + SBI)	The ultimate integration of digital twin modeling and SBI to create bio-silico hybrid systems capable of learning, adaptation, and cognitive processing Panuccio et al. (2016); Smirnova and Hartung (2024).

S2. Final comparison of major neuromorphic architectures*

Aspect	Loihi Davies et al. (2018)	TrueNorth Akopyan et al. (2015)	SpiNNaker Furber et al. (2012)	DYNAP-SE2 Richter et al. (2024)	BrainScaleS2 Pehle et al. (2022)	Tianjic Deng et al. (2020)
Clocking	Fully async; event-driven (no global clock)	Mixed async–sync cores; event-driven routing	Asynchronous interconnect; no global clock	Fully async mixed-signal design	1,000× accelerated analog time evolution; digital processors for control	Hybrid ANN/SNN; sync cores + async mesh routing
Communication	2D-mesh NoC; AER-based spike routing	2D-mesh; asynchronous router; AER routing; merge–split blocks for multi-chip scaling	Packet-based NN, P2P, MC, FR packets; 2D torus NoC	On-chip AER; 1024 neurons per chip	On-chip event-routing network + custom off-chip link protocol	2D-mesh NoC for spikes and ANN data
Neuron model	Digital LIF; multi-compartment; stochastic options	Augmented LIF; stochastic + deterministic, 1–3 neurons emulate 20 spiking behaviors	Point neuron model; 1000 neurons/core real-time	Thresholded I&F and AdEx; sub-threshold analog	Analog adaptive Exponential IF (AdEx)	Hybrid: binary SNN neurons; 8-bit ANN activations
Synapse type	On-chip SRAM; 1–9 bit weights; 16 MB total	64k synaptic crossbar (256×256), four weight types (integer)	Excitatory & inhibitory with scalar weights	64 synapses; 4-bit weight; 2-bit delay; STP depression	SRAM-stored weights; current & conductance-based synapses	SRAM per core; shared ANN/SNN weights
On-chip learning	Yes: microcoded STDP & RL plasticity	No: inference only; offline training	Yes: in software on ARM cores	Local short-term plasticity + homeostasis; no long-term plasticity	Yes: PPUs for STDP, R-STDP, homeostatic, structural plasticity	No: inference only; pre-trained weights
Energy per syn-op	20–25 pJ; STDP ∼120 pJ	400 GSOPS/W (max); 46 GSOPS/W at 65 mW for typical workload	2,200 MIPS/W (1,036,800 cores × 200 MHz / 90 kW power)	150 pJ (I&F); 300 pJ (AdEx) at 80 Hz	N/A	1.28 TOPS/W (ANN); 649 GSyOPS/W (SNN) at 300 MHz, 0.85 V
Biological realism	Spiking, local plasticity, moderate realism	Spiking dynamics approximate biological behavior; 1–3 neurons replicate 20 Izhikevich behaviors	Real-time; simplified neuron/synapse models	Modeled Alpha-EPSCs, NMDA gating, AMPA diffusion, adaptation	High: multi-compartment analog neurons + biologically plausible learning	Hybrid SNN/ANN; spiking dynamics + ANN activations

*NoC = Network-on-Chip; AER = Address-Event Representation; P2P = Point-to-Point; MC = Multicast; FR = Fixed-Route; LIF = Leaky Integrate-and-Fire; I&F = Integrate-and-Fire; AdEx = Adaptive Exponential Integrate-and-Fire; R-STDP = Reward-modulated STDP; RL = Reinforcement Learning; PPU = Plasticity Processing Unit; EPSC = Excitatory Post-Synaptic Current; AMPA = α-amino-3-hydroxy-5-methyl-4-isoxazolepropionic acid; TOPS/W = Tera-Operations per Second per Watt; GSOPS/W = Giga Synaptic Operations per Second per Watt; GSyOPS/W = Giga *Spiking* Synaptic Operations per Second per Watt; MIPS/W = Million Instructions per Second per Watt; SRAM = Static RAM; ARM = Advanced RISC Machine.
